# Predictors of admission to an assertive outreach service for psychosis in Lebanon

**DOI:** 10.1371/journal.pgph.0001428

**Published:** 2022-12-28

**Authors:** Ghida Kassir, Samer El Hayek, Raghid Charara, Michele Cherro, Hala Itani, Joseph El Khoury

**Affiliations:** 1 Department of Psychiatry, American University of Beirut, Beirut, Lebanon; 2 Department of Psychiatry and Behavioral Sciences, University of Miami Miller School of Medicine, Miami, Florida, United States of America; 3 Center for Behavioral Health, Neurological Institute, Cleveland Clinic, Cleveland, Ohio, United States of America; 4 Department of Psychiatry and Behavioral Health, American Hospital Dubai, Dubai, United Arab Emirates; University of Washington, UNITED STATES

## Abstract

Schizophrenia is a chronic, debilitating mental illness that contributes significantly to the global burden of disease. Assertive outreach treatment for patients with schizophrenia and psychotic disorders has been implemented to improve treatment adherence and outcomes. The suitability of this model of care outside the western context has not been fully established. We describe the characteristics of 45 patients enrolled in the Psychosis Recovery Outreach Program (PROP), a program developed at a leading psychiatric facility in Lebanon. We collected twelve-month data for patients and used logistic regression models to identify predictor variables for enrollment in the service compared to those receiving standard treatment. Patients were mostly males (77.8%), younger than 39 years (80%), of college or higher education (68.2%), and diagnosed with schizophrenia (46.7%) or schizoaffective disorder (48.9%). About one-quarter (22.7%) had a comorbid cannabis use disorder. A majority received more than one oral antipsychotic (75.6%) while half (51.1%) were maintained on a long-acting injectable (LAI) antipsychotic. The following variables were significant predictors of enrollment in PROP: having a comorbid cannabis use disorder (OR 2.83 [1.25 – 6.37]), being prescribed a LAI antipsychotic (OR 9.99 [4.93-20.24]) or more than one oral antipsychotic (OR 4.57 [2.22-9.39]), visiting the emergency department more than once (OR 8.7 [2.64-28.68]), and admission to the psychiatry unit (OR 13.91 [3.17-60.94]). In addition, those following up in PROP were younger and less likely to be in the oldest age group (over 54 years) [OR 0.11 (0.01-0.93)], less likely to be females (OR 0.39 [0.18-0.81]), and less likely to be diagnosed with “other psychotic disorder” as compared to schizophrenia (OR 0.14 [0.03 – 0.62]). Our findings highlight that the assertive outreach model of care is applicable to its target population in the context of psychiatric care in Lebanon, namely young individuals with psychosis, higher comorbidities and a severe course of illness.

## Introduction

Schizophrenia is a chronic and debilitating mental illness characterized by positive symptoms, negative symptoms, cognitive distortions, and disorganized behaviors [1]. The worldwide prevalence of schizophrenia is estimated at around 1%. With a typical age of onset in late adolescence and early adulthood, it leads to significant disability and reduction in quality of life over the years [2]. According to the Global Burden of Disease study, cases increased from 14.2 million in 1990 to 23.6 million in 2019. This contributes to 12.2% disability-adjusted life-years globally [3]. Untreated or under-treated schizophrenia often translates into a cascade of negative outcomes including reduced economic productivity, family instability, disrupted education, high medical morbidity, early mortality and homelessness [4]. In 2012, the worldwide economic burden of schizophrenia reached an estimated 56 billion dollars. This includes indirect costs, direct healthcare costs, direct non-medical costs, and social costs [5].

The effective treatment of schizophrenia faces multiple challenges, some of which are inherent to the disease. Additional complications include psychiatric comorbidities, such as substance use and mood disorders, known to worsen prognosis [6] and increase the risk of suicide [7]. Another concern is the ubiquity of medical comorbidity, particularly of a cardio-metabolic nature, which is responsible for morbidity and mortality. This necessitates that patients with schizophrenia receive regular medical monitoring and treatment [7]. Despite advances in psychopharmacology, a major challenge remains, namely medication non-adherence, primarily blamed for poor disease outcomes [8]. The rate of medication non-adherence ranges between 37% and 74% resulting in increased hospitalization rates, repeated relapses, and subsequent greater resource usage [9]. Therefore, close follow-up of patients with schizophrenia is deemed a priority to address poor prognosis [10].

Over the years, a number of interventions have been promoted with the aim to improve prognosis in schizophrenia. These include pharmacologic interventions such as the early use of LAI antipsychotics and psychosocial ones such as the implementation of shared-decision making programs in a recovery-oriented mental healthcare system [11]. Assertive outreach treatment (AOT) is a service level intervention that targets patients with severe mental illness (SMI) who do not engage effectively with traditional services [12]. This distinct model of care originated in the United States of America (USA) as early as 1967 under the Assertive Community Treatment (ACT) name to address identified gaps in the continuity of care of SMI patients following discharge from hospital [13]. It was subsequently adapted by other health systems while retaining the core elements and spirit. The American and Australian application focused on reduction in hospitalization and greater social inclusion for patients [14]. Australian studies showed conclusively that patients receiving assertive care had fewer hospitalizations, shorter hospital stays, and a better quality of life [15]. In the United Kingdom (UK), an additional measured outcome was risk reduction towards self and the public [14]. A variation on assertive community care dedicated to early psychosis resulted in more sustained contact with services and resulted in lower rates of symptomatic relapse and readmission rates [16].

In Lebanon, a low middle income country, the prevalence of schizophrenia is estimated at 50,000 individuals for a population of five million [17]. Mental health services are historically under-resourced, with an estimated 1.26 psychiatrists per 100,000 population [18], well below international recommendations [19]. Specialist inpatient, acute and residential, services consist respectively of 28.52 beds and 1.5 beds per 100,000 population with a 97% occupancy rate [18]. It is in this context that the first specialist community service for patients diagnosed with a psychotic disorder was established. The Psychosis Recovery Outreach Program (PROP), staffed by a multidisciplinary team that applies principles of early intervention and assertive outreach, was initiated in February 2016 at the American University of Beirut Medical Center (AUBMC) [17].

The aim of this study is to identify and analyze clinical and demographic variables associated with patient enrollment in PROP, out of a typical clinical population attending a psychiatric outpatient department. The wide applicability and efficacy of the assertive outreach model of care in low- and middle-income countries has not been yet demonstrated. Identifying the population likely to engage and benefit from it is an early step in this direction. We hypothesize that patients following up in PROP and benefiting from the services of this outreach program are likely to be individuals suffering from severe mental illnesses with a high risk of relapse and worse prognostic factors.

## Methods

### Ethics statement

The study was approved by the Institutional Review Board (IRB) committee of AUBMC under the following approval number (BIO-2020-0468).

### Study design and study population

This retrospective study was conducted at AUBMC, a major tertiary care center located in the capital Beirut.

The subjects consisted of patients who presented to the outpatient psychiatry department and were following up in PROP from 1^st^ November 2018 to 1^st^ November 2019. Clinical data was extracted up until 1^st^ November 2020, to ensure that a minimum of twelve months was covered for all patients. Inclusion criteria included having an age of 18 years and above at time of first point of care and a primary diagnosis of psychosis according to the International Classification of Diseases 10 (ICD-10). Diagnoses included psychotic disorder with hallucinations due to known physiologic condition (F06.0), psychotic disorder with delusions due to known physiologic condition (F06.2), other psychoactive substance abuse with psychoactive substance-induced psychosis (F19.15), other psychoactive substance abuse, unspecified, with psychoactive substance-induced psychosis (F19.95), schizophrenia (F20), delusional disorders (F22), brief psychotic disorder (F23), shared psychotic disorder (F24), schizoaffective disorders (F25), other psychotic disorder not due to a substance or known physiological condition (F28), and unspecified psychosis not due to a substance or known physiological condition (F29). Additional non-classification specific diagnoses available on Epic Systems that included the terms “psychosis” and “schizophrenia” were also considered eligible. Patients whose diagnosis was changed to a non-psychotic disorder during the period of investigation were excluded. These clinical diagnoses were exclusively made by US and UK trained qualified psychiatrists based on clinical observations and interviews, past medical records, and collateral history.

### Data extraction

The data was retrieved and collected from the patients’ Electronic Health Records (EHR) available through EPIC Systems at AUBMC. Access to data without patients’ consent was authorized by the IRB. The extraction tool was developed by the team and included the following study variables: patients’ demographics (age, gender, education level, and employment status), ICD-10 diagnoses of psychotic illness (as previously stated), ICD-10 diagnoses of comorbid psychiatric disorders (bipolar disorder, depressive disorder, substance use disorder, personality disorder, anxiety disorder, obsessive-compulsive disorder (OCD), feeding and eating disorder, neurodevelopmental disorder, disruptive and conduct disorder, sexual dysfunction, sleep-wake disorder, dissociative disorder, elimination disorder, somatic symptoms disorder, neurocognitive disorder, and trauma-related disorder), ICD-10 diagnoses of comorbid medical disorders (immune system disease, neoplasms, endocrine, nutritional, and metabolic disorder, nervous system disorder, circulatory system disease, respiratory system disease, digestive system disease, and others), substances used throughout the follow-up period (alcohol, hallucinogens, cannabis, inhalants, sedatives/hypnotics, opiates, stimulants, and tobacco), family history of mental illness, antipsychotic treatment (period of treatment, number of antipsychotics prescribed throughout the follow-up period, name, generation, dosage, and formulation), and unscheduled use of psychiatric services (unscheduled use of phone-based nursing services, visits to the emergency department, and hospitalizations to the inpatient psychiatry ward). To test the inter-rater reliability of the extraction tool, initially, four reviewers (GK, SEH, HI, and MC) independently extracted data for the same 10 patients and entered it into a standardized table that included all the variables. A first meeting was held to compare the data extracted by the four reviewers. All disagreements were resolved after discussion with a fifth reviewer (JEK) and the extraction tool was adjusted accordingly. The same four reviewers independently extracted data for 10 other patients then held an additional meeting to compare and align results.

### Predictor and outcome variables

The primary outcome of the study was defined as enrollment in PROP (yes/no). Clinical and demographic variables that are known to influence the course and prognosis of schizophrenia and hypothesized to correlate with enrollment in an assertive outreach type program were recorded. Those included age, gender, education level, employment status, type of psychotic illness, number of psychiatric comorbidities (0, 1, ≥2), comorbid mood disorder, comorbid medical disorder, family history of psychiatric illness, comorbid alcohol use disorder, comorbid cannabis use disorder, the prescription of a Long Acting Injectable (LAI) antipsychotic, the number of prescribed oral antipsychotics (1, >1), unscheduled use of phone-based nursing services (no calls, 1-2 calls, >2 calls), visits to the emergency department (no visits, 1 visit, >1 visit), hospitalizations to the inpatient psychiatry ward (none, 1 admission, >1 admission), and the use of any unscheduled service.

### Statistical analysis

Data were analyzed using Stata (Version 15.1). Descriptive data were presented to reflect the distribution of outcome and predictor variables. Logistic regression models were run to analyze the association between PROP enrollment and measured predictor variables (socio-demographic variables, psychiatric predictors, medication prescriptions, and use of services).

## Results

### General characteristics of participants

330 subjects were included in our final sample, which represents all active cases with a diagnosis of psychosis at the psychiatry department between November 2018 and November 2020. The general characteristics of the participants are presented in **Table 1**. Forty-five patients were enrolled in PROP (13.64%). Males were slightly over-represented in both the total (60.4%) and PROP (77.8%) samples. Most participants following in PROP were younger than 39 years old (80%) and had achieved a college degree or some form of higher education (68.2%). There was an almost equal distribution between participants who were never employed and those who were previously or currently employed (51.5% vs. 48.5%, respectively.) Schizophrenia (46.7%) and schizoaffective disorder (48.9%) accounted for the overwhelming majority of diagnoses. About one-third of PROP patients (33.3%) had a comorbid medical disorder and 9% had a comorbid mood disorder. In addition, 13.3% and 22.7% of patients had comorbid alcohol and cannabis use disorders, respectively. Otherwise, most of the sample (76%) did not have a comorbid psychiatric disorder. In terms of treatment, the majority of patients (75.6%) were prescribed more than one oral antipsychotic and half (51.1%) a LAI antipsychotic.

**Table 1:** General characteristics of patients with a psychotic disorder seen at the outpatient psychiatry department at AUBMC (n = 330).

	Non-PROP (285)	PROP (45)	Total (330)
	% (n)	% (n)	% (n)
***SOCIODEMOGRAPHIC VARIABLES***			
**Age (years)**			
< 25	17.1 (49)	22.2 (10)	17.9 (59)
25 – 39	27.2 (106)	57.8 (26)	40.0 (132)
40 – 54	30.5 (87)	17.8 (8)	28.8 (95)
> 54	15.1 (43)	2.2 (1)	13.3 (44)
**Sex**			
Male	57.6 (163)	77.8 (35)	60.4 (198)
Female	42.4 (120)	22.2 (10)	39.6 (130)
**Education**			
High school or below	38.1 (51)	31.8 (7)	37.2 (58)
College or above	61.9 (83)	68.2 (15)	62.8 (98)
**Employment**			
Never employed	47.4 (83)	51.5 (17)	48.1 (100)
Previously/currently employed	52.6 (92)	48.5 (16)	51.9 (108)
***PSYCHIATRIC VARIABLES***			
**Type of psychotic illness**			
Schizophrenia	43.2 (123)	46.7 (21)	43.6 (144)
Schizoaffective disorder	27.7 (79)	48.9 (22)	30.6 (101)
Other psychotic disorder*	29.1 (83)	4.4 (2)	25.8 (85)
**Number of psychiatric comorbidities**			
0	66.0 (188)	75.6 (34)	67.3 (222)
1	24.2 (69)	13.3 (6)	22.7 (75)
≥ 2	9.8 (28)	11.1 (5)	10 (33)
**Comorbid mood disorder**			
No	87.7 (250)	91.1 (41)	88.2 (291)
Yes	12.3 (35)	8.9 (4)	11.8 (39)
**Comorbid medical disorder**			
No	54.4 (155)	66.7 (30)	56.1 (185)
Yes	45.6 (130)	33.3 (15)	43.9 (145)
**Family history of psychiatric illness**			
No	79.8 (202)	69.2 (27)	78.4 (229)
Yes	20.2 (51)	30.8 (12)	21.6 (63)
**Comorbid alcohol use disorder**			
No	89.9 (248)	86.7 (39)	89.4 (287)
Yes	10.1 (28)	13.3 (6)	10.6 (34)
**Comorbid cannabis use disorder**			
No	90.6 (250)	77.3 (34)	88.8 (284)
Yes	9.4 (26)	22.7 (10)	11.3 (36)
***PSYCHOTROPIC VARIABLES***			
**LAI prescription**			
No	90.5 (258)	48.9 (22)	84.9 (280)
Yes	9.5 (27)	51.1 (23)	15.2 (50)
**Number of prescribed oral AP**			
1	59.7 (170)	24.4 (11)	54.9 (181)
> 1	40.4 (115)	75.6 (34)	45.2 (149)
***SERVICE USE VARIABLES*** **			
**Nursing services (calls)**			
No calls	51.9 (148)	57.8 (26)	52.7 (174)
1-2 calls	24.6 (70)	17.8 (8)	23.6 (78)
> 2 calls	23.5 (67)	24.4 (11)	23.6 (78)
**ED services**			
No ED visits	91.6 (261)	66.7 (30)	88.2 (291)
1 ED visit	6.3 (18)	20.0 (9)	8.2 (27)
> 1 ED visit	2.1 (6)	13.3 (6)	3.6 (12)
**Inpatient admissions**			
None	93.7 (267)	71.1 (32)	90.6 (299)
1 admission	5.3 (15)	17.8 (8)	7.0 (23)
> 1 admission	1.1 (3)	11.1 (5)	2.4 (8)
**Any service**			
No	49.8 (142)	53.3 (24)	50.3 (166)
Yes	50.2 (143)	46.7 (21)	49.7 (164)

* schizophreniform disorder, brief psychotic disorder, delusional disorder, substance-induced psychosis, unspecified psychotic disorder, psychotic disorder due to a general medical condition

** over the study period

### Predictors of PROP enrollment

We found significant associations between several variables and enrollment in PROP (**Table 2**). These variables included age, gender, diagnosis, having comorbid cannabis use disorder, being prescribed a LAI antipsychotic, being prescribed more than one oral antipsychotic, and using unscheduled mental health services, specifically visiting the emergency department and being admitted to the psychiatry unit.

**Table 2:** Adjusted odds ratio with 95% confidence intervals of the association between PROP enrollment and predictors of enrollment.

	PROP membership (no vs yes)	
	OR (95% CI)	p-value
***SOCIODEMOGRAPHIC VARIABLES***		
**Age (years)**		
< 25	1.00	
25 – 39	1.20 (0.54 – 2.69)	0.65
40 – 54	0.45 (0.17 – 1.22)	0.12
> 54	**0.11 (0.01 – 0.93**)	**0.04**
**Sex**		
Male	1.00	
Female	**0.39 (0.18 – 0.81)**	**0.01**
**Education**		
High school or below	1.00	
College or above	1.32 (0.50 – 3.45)	0.58
**Employment**		
Never employed	1.00	
Previously/currently employed	0.85 (0.40 – 1.79)	0.67
***PSYCHIATRIC VARIABLES***		
**Type of psychotic illness**		
Schizophrenia	1.00	
Schizoaffective disorder	1.63 (0.84 – 3.16)	0.15
Other psychotic disorder*	**0.14 (0.03 – 0.62)**	**0.01**
**Number of psychiatric comorbidities**		
0	1.00	
1	0.48 (0.19 – 1.20)	0.12
≥ 2	0.99 (0.36 – 2.74)	0.98
**Comorbid mood disorder**		
No	1.00	
Yes	0.70 (0.24 – 2.06)	0.51
**Comorbid medical disorder**		
No	1.00	
Yes	0.60 (0.31 – 1.16)	0.13
**Family history of psychiatric illness**		
No	1.00	
Yes	1.76 (0.83 – 3.71)	0.14
**Comorbid alcohol use disorder**		
No	1.00	
Yes	1.36 (0.53 – 3.50)	0.52
**Comorbid cannabis use disorder**		
No	1.00	
Yes	**2.83 (1.25 – 6.37)**	**0.01**
***PSYCHOTROPIC VARIABLES***		
**LAI prescription**		
No	1.00	
Yes	**9.99 (4.93 – 20.24)**	**<0.001**
**Number of prescribed oral AP**		
1	1.00	
> 1	**4.57 (2.22 – 9.39)**	**<0.001**
***SERVICE USE VARIABLES*** **		
**Nursing services (calls)**		
No calls	1.00	
1-2 calls	0.65 (0.28 – 1.51)	0.32
> 2 calls	0.93 (0.44 – 2.00)	0.86
**ED services**		
No ED visits	1.00	
1 ED visit	**4.35 (1.80 – 10.54)**	**0.001**
> 1 ED visit	**8.7 (2.64 – 28.68)**	**<0.001**
**Inpatient admissions**		
None	1.00	
1 admission	**4.45 (1.75 – 11.31)**	**0.002**
> 1 admission	**13.91 (3.17 – 60.94)**	**<0.001**
**Any service**		
No	1.00	
Yes	0.87 (0.46 – 1.63)	0.66

Bolded values denote statistical significance (p < 0.005).

* schizophreniform disorder, brief psychotic disorder, delusional disorder, substance-induced psychosis, unspecified psychotic disorder, psychotic disorder due to a general medical condition

** over the study period

Compared to patients receiving standard care, those in PROP were younger and significantly less likely to be in the oldest age group (over 54 years) [OR 0.11 (0.01-0.93)]. Males were two to three-fold more likely to be enrolled in PROP compared to females (OR 0.39 [0.18-0.81]). Those in the PROP sample were less likely to be diagnosed with “other psychotic disorder” as compared to schizophrenia (OR 0.14 [0.03 – 0.62]). They were also three times more likely to have a diagnosis of cannabis use disorder (OR 2.83 [1.25 – 6.37]). In terms of treatment, those following up in PROP were almost 5 times more likely to have been prescribed more than one oral antipsychotic (OR 4.57 [2.22 – 9.39]) and around 10 times more likely to have been prescribed a LAI antipsychotic (OR 9.99 [4.93 – 20.24]). As for service use, PROP patients were 8 times more likely to visit the emergency department more than once (OR 8.7 [2.64 – 28.68]) and 14 times more likely to be admitted to the psychiatry ward (OR 13.91 [3.17 – 60.94]).

## Discussion

In our study, significant differences were noted in a number of demographic and clinical variables between patients under the assertive outreach model (PROP) versus those receiving standard outpatient care. PROP patients tended to be younger males with a diagnosis of schizophrenia and have a comorbid cannabis use disorder. They also were more likely to be prescribed a LAI antipsychotic and/or be prescribed more than one oral antipsychotic. Their use of services was higher, including attendance to the emergency department and admission to the psychiatry inpatient unit. These results seem to suggest that patients with the worst prognostic factors are being referred and included in the program. This is in line with its intended mission. As the literature supports, this group has the most to benefit from assertive community treatment programs [20, 21]. The young age at enrollment could be attributed to several factors. First, older adults with psychotic illnesses tend to have less severe active symptomatology. Indeed, some studies showed reduction in the number and intensity of positive symptoms in patients with schizophrenia over time due to the natural course of the illness [22]. Aging was associated with better psychosocial functioning, decreased positive psychotic symptoms, less substance use, lower psychiatric hospitalization rates, and improved overall mental health [23]. Some studies even looked at the need to decrease antipsychotic dosages or eventually discontinue medications in older adults with schizophrenia, due to their multiple side effects and possibly limited effectiveness in this population [22]. The apathy and amotivation associated with negative symptoms could explain why this group of patients were less likely to be enrolled in PROP despite referral. A systematic review published in 2019 on the longitudinal course of schizophrenia across the lifespan showed that negative symptoms tend to increase or remain relatively constant despite treatment [24]. Additionally, older adults with psychotic illnesses may shy away from seeking help due to stigma while clinicians and care providers may also be satisfied with maintaining some form of stability even at the expense of functionality. One study suggested that schizophrenia in older adults is associated with a double social stigma [25], with this population receiving less recognition, treatment, rehabilitation, and engagement in society [26].

Males were more likely to be enrolled in PROP than females, who are already under-represented in the overall sample. Research shows that schizophrenia and non-affective psychoses are more common in males who have a worse prognosis, poorer psychosocial functioning, and reduced response to treatment compared to females [27]. One can hypothesize that males with psychosis are more likely to require assertive outreach due to illness severity. Another angle is that both the manifestation of the disease in males and the social perception of it is also linked to gender roles expectations, such as economic productivity or the concern over more disruptive and even violent behavior. In more conservative societies, males tend to be the primary breadwinners and have a wider social network to preserve.

In terms of diagnoses, patients with a diagnosis of “other psychotic disorder” were less likely to be enrolled in PROP. Multiple studies have attempted to compare several outcomes between patients with schizophrenia and other psychotic disorders, whether affective or non-affective in nature. One study found that patients with schizophrenia had more severe impairments, specifically in instrumental work functioning, and showed more persistent psychopathology and problems in functioning than patients with other psychotic disorders [28]. Reaching a diagnosis of schizophrenia, especially within the first year of presentation, usually implies clinical certainty on the part of the treating psychiatrist that could be explained by active first-rank symptoms and a more severe illness course. The comorbidity of a cannabis use disorder was strongly associated with enrollment in PROP. Other substance use disorders were too rare in our sample to allow comparison. In a meta-analysis that looked at cannabis use in psychotic disorders, continued use after onset of psychosis predicted higher relapse rates, longer hospital admissions, and more severe positive symptoms [29]. The relative age of the population in treatment where cannabis use is prevalent may also account for this number.

Patients enrolled in PROP were more likely to be prescribed LAI antipsychotics than those in standard care. Non-adherence to antipsychotics is notoriously high in psychotic disorders and responsible for early relapse and poor prognosis [30–32]. One crucial contributing factor is the absence of insight inherent to the psychotic process [33]. LAI antipsychotics are gaining ground, in particular in early psychosis, due to advantages over oral formulations. These include more stable blood concentrations, consistent bioavailability, an improved pharmacokinetic profile, and predictable medication adherence [10]. A more assertive approach to treatment may indicate a general propensity to use LAI antipsychotics due to the evidence surrounding them. In parallel, patients enrolled in PROP possibly have a severe illness course and limited insight into their condition, limiting the efficacy of any oral medication. This second hypothesis is supported by the finding that they were also more likely to be prescribed more than one oral antipsychotic. Polypharmacy generally indicates either a more resistant illness or poor medication compliance often leading to the use of LAI [34]. In fact, despite all the measures put in place, patients enrolled in PROP were still more likely to use unscheduled mental health services, including visits to the emergency department and admission to the psychiatry unit. Several studies have shown that patients use unscheduled services due to illness exacerbations and the urgent need to follow up, [35]. For example, one study that looked at the use of unscheduled mental health services in a cohort of patients with psychotic disorders following up in outpatient clinics showed that despite regular follow ups with mental health providers, half of the patients had at least one unscheduled clinical contact over 12 months, including phone-based nursing services and visits to the emergency departments, and up to 1 in 10 patients was still admitted to the psychiatry unit [36].

Economic constraints in low- and middle-income countries (LMIC) challenge health care services to identify efficient and cost-effective methods of delivering mental health care. Few of these countries have implemented formal outreach community services for patients with severe mental disorders, which include psychotic disorders. Examples of such services are the community engagement mental health (CEMH) model in Jamaica [37], the “Care at Doorsteps” (CADS) initiative in India [38] and the Chinese Assertive Community Treatment (ACT) in China [39]. These outreach programs all share similarities with PROP; from adopting a task sharing model that shifts key elements of care delivery from the psychiatrist to trained nurse practitioners, psychiatry residents, psychologists, social workers, to shifting clinical interaction from the clinic to the community when possible [17, 37]. In addition, one common goal of these outreach programs is the emphasis on social functioning and inclusion as a primary outcome rather than symptom relief alone [37]. One similarity between Lebanon and China is the prominence of family support; in both countries, most patients live with their families and families are by default included in the community care model [39]. This stresses the importance of allocating resources for regular psychoeducation of families, which has been shown to help strengthen social support, improve adherence to antipsychotics and decrease admission rates [40, 41]. The assertive model of care demonstrated to be effective is expected to include a full range of accommodation arrangements, medication optimization, regular psychiatric evaluations, adequate in-patient support, rehabilitation activities, psychosocial support, and rapid crisis response [42]. The absence of a number of these elements is often a major limiting factor for effectiveness despite the therapeutic effort put forward by clinicians. Specialist outreach services in LMIC often struggle to incorporate them in care packages in the absence of effective integration of advanced health and social services. Until such integration is possible, an adapted model of assertive treatment is needed for fragile healthcare settings.

### Limitations

Our study has several limitations. Our sample size is relatively small, restricting the power to elicit more associations. Participants were recruited from one healthcare center in Lebanon. Data were retrieved retrospectively from clinical charts not intended for research purposes; this was reflected in missing data and the absence of objective measurement scales that could have provided additional insight relevant to the study. Future studies would benefit from a larger population spanning multiple hospitals or services, along with the use of a more comprehensive database and standardized rating tools for the assessment of variables.

## Conclusion

PROP was the first community treatment program to use the principles of assertive outreach in Lebanon. Its establishment was an important step in addressing the public health burden presented by psychotic disorders. Identifying the characteristics of patients who received care under the program helps understand the applicability of the assertive outreach model to the context of Lebanon, a low-middle income country with chronic economic and political challenges. Locally driven initiatives that adapt established models in developed healthcare systems to low resources health economies provide an opportunity to deliver cost-effective care to high morbidity population.

## Supporting information

S1 Data The table contains the raw data which was used to generate the results in the manuscript and is freely available to other researchers. Legend: **NOS**: not otherwise specified, **OCD**: obsessive-compulsive disorder, **AP**: antipsychotic, **LAI**: long-acting injectable, **ED**: emergency department, **PROP**: Psychosis Recovery Outreach Program, **N/A**: not applicable.
(XLSX)

## References

[1] Sicilia V, Del Bello V, Verdolini N, Tortorella A, Moretti P. Oral versus long-acting injectable antipsychotics: hospitalisation rate of psychotic patients discharged from an Italian Psychiatric Unit. Psychiatria Danubina. 2017;29(Suppl 3):333–40. Epub 2017/09/28. . 28953786

[2] Uhlmann C, Kaehler J, Harris MS, Unser J, Arolt V, Lencer R. Negative impact of self-stigmatization on attitude toward medication adherence in patients with psychosis. Journal of psychiatric practice. 2014;20(5):405–10. Epub 2014/09/17. doi: 10.1097/01.pra.0000454787.75106.ae . 25226204

[3] Global, regional, and national burden of 12 mental disorders in 204 countries and territories, 1990–2019: a systematic analysis for the Global Burden of Disease Study 2019. The Lancet Psychiatry. 2022;9(2):137–50. doi: 10.1016/S2215-0366(21)00395-3 35026139 PMC8776563

[4] Barbato A, Initiative WHONfMH, World Health Organization. Division of Mental H, Prevention of Substance A. Schizophrenia and public health / Angelo Barbato. Geneva: World Health Organization; 1997.

[5] Tinelli M, Kanavos P. Cost and impact of non-treating severe mental illnesses (SMIs): The case study of schizophrenia 2015.

[6] Abdullah HM, Azeb Shahul H, Hwang MY, Ferrando S. Comorbidity in Schizophrenia: Conceptual Issues and Clinical Management. Focus (American Psychiatric Publishing). 2020;18(4):386–90. Epub 2020/12/22. doi: 10.1176/appi.focus.20200026 ; PubMed Central PMCID: PMC7725147. 33343250 PMC7725147

[7] Mitchell AJ, Vancampfort D, Sweers K, van Winkel R, Yu W, De Hert M. Prevalence of metabolic syndrome and metabolic abnormalities in schizophrenia and related disorders--a systematic review and meta-analysis. Schizophrenia bulletin. 2013;39(2):306–18. Epub 2011/12/31. doi: 10.1093/schbul/sbr148 ; PubMed Central PMCID: PMC3576174. 22207632 PMC3576174

[8] Acosta FJ, Hernández JL, Pereira J, Herrera J, Rodríguez CJ. Medication adherence in schizophrenia. World journal of psychiatry. 2012;2(5):74–82. Epub 2013/11/01. doi: 10.5498/wjp.v2.i5.74 ; PubMed Central PMCID: PMC3782179. 24175171 PMC3782179

[9] Patel KR, Cherian J, Gohil K, Atkinson D. Schizophrenia: overview and treatment options. P & T: a peer-reviewed journal for formulary management. 2014;39(9):638–45. Epub 2014/09/12. ; PubMed Central PMCID: PMC4159061. 25210417 PMC4159061

[10] Kishi T, Oya K, Iwata N. Long-acting injectable antipsychotics for the prevention of relapse in patients with recent-onset psychotic disorders: A systematic review and meta-analysis of randomized controlled trials. Psychiatry research. 2016;246:750–5. Epub 2016/11/20. doi: 10.1016/j.psychres.2016.10.053 . 27863801

[11] Lo WA-L, Ki-Yan Mak D, Ming-Cheuk Wong M, Chan O-W, Mo-Ching Chui E, Wai-Sau Chung D, et al. Achieving better outcomes for schizophrenia patients in Hong Kong: Strategies for improving treatment adherence. CNS Neurosci Ther. 2021;27 Suppl 1:12–9. doi: 10.1111/cns.13375 . 33555616 PMC7869929

[12] Stein LI, Test MA, Marx AJ. Alternative to the hospital: a controlled study. The American journal of psychiatry. 1975;132(5):517–22. Epub 1975/05/01. doi: 10.1176/ajp.132.5.517 164129

[13] MCADAM M WR IGHT N. A review of the literature considering the role of mental health nurses in assertive outreach. 2005;12(6):648–60. doi: 10.1111/j.1365-2850.2005.00889.x 16336589

[14] Harvey C, Killaspy H, Martino S, White S, Priebe S, Wright C, et al. A comparison of the implementation of assertive community treatment in Melbourne, Australia and London, England. Epidemiol Psychiatr Sci. 2011;20(2):151–61. doi: 10.1017/s2045796011000230 . 21714362

[15] McGorry PD, Edwards J, Mihalopoulos C, Harrigan SM, Jackson HJ. EPPIC: an evolving system of early detection and optimal management. Schizophrenia bulletin. 1996;22(2):305–26. Epub 1996/01/01. doi: 10.1093/schbul/22.2.305 . 8782288

[16] Craig TK, Garety P, Power P, Rahaman N, Colbert S, Fornells-Ambrojo M, et al. The Lambeth Early Onset (LEO) Team: randomised controlled trial of the effectiveness of specialised care for early psychosis. BMJ (Clinical research ed). 2004;329(7474):1067. Epub 2004/10/16. doi: 10.1136/bmj.38246.594873.7C ; PubMed Central PMCID: PMC526115. 15485934 PMC526115

[17] El-Khoury J, Ghazzaoui R, Ahmad A. Introducing Specialist Integrated Mental Health Care in Lebanon: The Psychosis Recovery Outreach Program. Psychiatr Serv. 2018;69(7):738–40. doi: 10.1176/appi.ps.201800018 . 29540116

[18] Health MoP. WHO–AIMS Report on Mental Health System in Lebanon Beirut, Lebanon 2015 [cited 2022 July 7]. Available from: https://www.moph.gov.lb/en/Pages/0/9109/who-aims-report-on-mental-health-system-in-lebanon.

[19] World Health O. Health workforce requirements for universal health coverage and the Sustainable Development Goals. (Human Resources for Health Observer, 17). Geneva: World Health Organization; 2016 2016.

[20] Yee MR, Espiridon E, Oladunjoye AO, Millsaps U, Harvey N, Vora AH. The Use of Clozapine in the Serious Mental Illness Patients Enrolled in an Assertive Community Treatment Program. Cureus. 2021;13(5):e15238. doi: 10.7759/cureus.15238 ; PubMed Central PMCID: PMC8232999. 34188983 PMC8232999

[21] Thoegersen MH, Morthorst BR, Nordentoft M. Assertive community treatment versus standard treatment for severely mentally ill patients in Denmark: a quasi-experimental trial. Nord J Psychiatry. 2019;73(2):149–58. doi: 10.1080/08039488.2019.1576765 . 30894038

[22] Jeste DV, Maglione JE. Treating older adults with schizophrenia: challenges and opportunities. Schizophrenia bulletin. 2013;39(5):966–8. Epub 2013/04/05. doi: 10.1093/schbul/sbt043 ; PubMed Central PMCID: PMC3756792. 23552180 PMC3756792

[23] Jeste DV, Wolkowitz OM, Palmer BW. Divergent trajectories of physical, cognitive, and psychosocial aging in schizophrenia. Schizophrenia bulletin. 2011;37(3):451–5. Epub 2011/04/21. doi: 10.1093/schbul/sbr026 ; PubMed Central PMCID: PMC3080682. 21505111 PMC3080682

[24] Heilbronner U, Samara M, Leucht S, Falkai P, Schulze TG. The Longitudinal Course of Schizophrenia Across the Lifespan: Clinical, Cognitive, and Neurobiological Aspects. Harvard review of psychiatry. 2016;24(2):118–28. Epub 2016/03/10. doi: 10.1097/HRP.0000000000000092 ; PubMed Central PMCID: PMC5079232. 26954596 PMC5079232

[25] Palmer BW, Heaton SC, Jeste DV. Older patients with schizophrenia: challenges in the coming decades. Psychiatric services (Washington, DC). 1999;50(9):1178–83. Epub 1999/09/09. doi: 10.1176/ps.50.9.1178 . 10478904

[26] Graham N, Lindesay J, Katona C, Bertolote JM, Camus V, Copeland JR, et al. Reducing stigma and discrimination against older people with mental disorders: a technical consensus statement. International journal of geriatric psychiatry. 2003;18(8):670–8. Epub 2003/08/02. doi: 10.1002/gps.876 . 12891632

[27] Ochoa S, Usall J, Cobo J, Labad X, Kulkarni J. Gender differences in schizophrenia and first-episode psychosis: a comprehensive literature review. Schizophrenia research and treatment. 2012;2012:916198. Epub 2012/09/12. doi: 10.1155/2012/916198 ; PubMed Central PMCID: PMC3420456. 22966451 PMC3420456

[28] Harrow M, Sands JR, Silverstein ML, Goldberg JF. Course and outcome for schizophrenia versus other psychotic patients: a longitudinal study. Schizophrenia bulletin. 1997;23(2):287–303. Epub 1997/01/01. doi: 10.1093/schbul/23.2.287 . 9165638

[29] Schoeler T, Monk A, Sami MB, Klamerus E, Foglia E, Brown R, et al. Continued versus discontinued cannabis use in patients with psychosis: a systematic review and meta-analysis. The lancet Psychiatry. 2016;3(3):215–25. Epub 2016/01/19. doi: 10.1016/S2215-0366(15)00363-6 . 26777297

[30] Montreuil TC, Cassidy CM, Rabinovitch M, Pawliuk N, Schmitz N, Joober R, et al. Case manager- and patient-rated alliance as a predictor of medication adherence in first-episode psychosis. Journal of clinical psychopharmacology. 2012;32(4):465–9. Epub 2012/06/23. doi: 10.1097/JCP.0b013e31825d3763 . 22722507

[31] Kim B, Lee SH, Yang YK, Park JI, Chung YC. Long-acting injectable antipsychotics for first-episode schizophrenia: the pros and cons. Schizophrenia research and treatment. 2012;2012:560836. Epub 2012/09/12. doi: 10.1155/2012/560836 ; PubMed Central PMCID: PMC3425805. 22966439 PMC3425805

[32] Zhornitsky S, Stip E. Oral versus Long-Acting Injectable Antipsychotics in the Treatment of Schizophrenia and Special Populations at Risk for Treatment Nonadherence: A Systematic Review. Schizophrenia research and treatment. 2012;2012:407171. Epub 2012/09/12. doi: 10.1155/2012/407171 ; PubMed Central PMCID: PMC3420751. 22966436 PMC3420751

[33] Jacob KS. Insight in Psychosis: An Indicator of Severity of Psychosis, an Explanatory Model of Illness, and a Coping Strategy. Indian journal of psychological medicine. 2016;38(3):194–201. Epub 2016/06/24. doi: 10.4103/0253-7176.183078 ; PubMed Central PMCID: PMC4904754. 27335513 PMC4904754

[34] Kreyenbuhl J, Marcus SC, West JC, Wilk J, Olfson M. Adding or switching antipsychotic medications in treatment-refractory schizophrenia. Psychiatric services (Washington, DC). 2007;58(7):983–90. Epub 2007/07/03. doi: 10.1176/ps.2007.58.7.983 ; PubMed Central PMCID: PMC3673548. 17602016 PMC3673548

[35] Langer S, Chew-Graham C, Hunter C, Guthrie EA, Salmon P. Why do patients with long-term conditions use unscheduled care? A qualitative literature review. Health & social care in the community. 2013;21(4):339–51. Epub 2012/09/27. doi: 10.1111/j.1365-2524.2012.01093.x ; PubMed Central PMCID: PMC3796281. 23009718 PMC3796281

[36] El Hayek S, Kassir G, Charara R, Genadry F, El Alayli A, El-Khoury J. Correlates of unscheduled and emergency clinical contact in a cohort of patients treated for psychosis. Psychiatry Research Communications. 2022;2(1):100024. 10.1016/j.psycom.2022.100024.

[37] Nelson D, Walcott G, Walters C, Hickling FW. Community Engagement Mental Health Model for Home Treatment of Psychosis in Jamaica. Psychiatr Serv. 2020;71(5):522–4. doi: 10.1176/appi.ps.201900063 . 32114944

[38] Basavaraju V, Murugesan M, Kumar CN, Gowda GS, Tamaraiselvan SK, Thirthalli J, et al. Care at door-steps for persons with severe mental disorders: A pilot experience from Karnataka district mental health program. Int J Soc Psychiatry. 2022;68(2):273–80. doi: 10.1177/0020764020983856 . 33356744

[39] Law SF, Luo X, Yao S, Wang X. Assertive Community Treatment in China - it is time for a made-in-China solution. Psychol Med. 2019;49(1):172–4. doi: 10.1017/S0033291718003094 . 30345937

[40] Armijo J, Mendez E, Morales R, Schilling S, Castro A, Alvarado R, et al. Efficacy of community treatments for schizophrenia and other psychotic disorders: a literature review. Front Psychiatry. 2013;4:116. doi: 10.3389/fpsyt.2013.00116 ; PubMed Central PMCID: PMC3793168. 24130534 PMC3793168

[41] Chien WT, Leung SF, Yeung FK, Wong WK. Current approaches to treatments for schizophrenia spectrum disorders, part II: psychosocial interventions and patient-focused perspectives in psychiatric care. Neuropsychiatr Dis Treat. 2013;9:1463–81. doi: 10.2147/NDT.S49263 ; PubMed Central PMCID: PMC3792827. 24109184 PMC3792827

[42] Dieterich M, Irving CB, Park B, Marshall M. Intensive case management for severe mental illness. Cochrane Database Syst Rev. 2010;(10):CD007906. doi: 10.1002/14651858.CD007906.pub2 ; PubMed Central PMCID: PMC4233116. 20927766 PMC4233116

